# Impact of fire on the macrofungal diversity in scrub jungles of south-west India

**DOI:** 10.1080/21501203.2016.1147090

**Published:** 2016-02-19

**Authors:** Ammatanda A. Greeshma, Kandikere R. Sridhar, Mundamoole Pavithra, Sudeep D. Ghate

**Affiliations:** Department of Biosciences, Mangalore University, Mangalagangotri, Mangalore574 199, Karnataka, India

**Keywords:** Mushrooms, diversity, abiotic factors, substrate, disturbance

## Abstract

Fortnightly survey in control and fire-impacted regions of scrub jungle of south-west coast of India during south-west monsoon (50 m^2^ quadrats up to 10 weeks) yielded 34 and 25 species of macrofungi, respectively. The species as well as sporocarp richness were the highest during the fourth week, while the diversity attained the highest during the second week in control region. In fire-impacted region, the species and sporocarp richness and diversity peaked at sixth week. Seven species common to both regions were *Chlorophyllum molybdites, Lepiota* sp., *Leucocoprinus birnbaumii, Marasmius* sp. 3, *Polyporus* sp., *Schizophyllum commune* and *Tetrapyrgos nigripes*. The overall sporocarp richness was higher in fire-impacted than in control region. The Jaccard’s similarity between regions was 13.5%, while fortnights of regions ranged from 0% (10th week) to 11.7% (eighth week). Control region showed single-species dominance by *Xylaria hypoxylon*, while multispecies dominance by *Cyathus striatus* and *Lentinus squarrosulus* in fire-impacted region. Except for air temperature, nine abiotic factors significantly differed between control and fire-impacted regions. The Pearson correlation was positive between species richness and phosphorus content in fire-impacted region (*r* = 0.696), while sporocarp richness was negatively correlated with pH in control region (*r* = −0.640). Economically viable species were 12 and 10 without overlap in control and fire-impacted regions, respectively.

## 1. Introduction

A wide variety of macrofungi serves as potential source of nutritional (edible), medicinal (bioactive compounds), agricultural (mutualists) and industrial (dyes and cosmetics) applications. Inventory of macrofungal inhabitants in different natural and human-influenced ecosystems broadens our knowledge on their usefulness. South-west coast of India is known for a variety of ecosystems, such as coastal sand dunes, mangroves, estuaries, bays, islands, freshwater marshes, sacred groves, scrub jungles and plantations. Unlike the Western Ghats, south-west coast of India embodies small to medium hilly ranges with lateritic scrub jungles owing to the impact of strong wind during south-west monsoon. Large expanses of these jungles are used for collection of leaf litter, green manure and firewood. Besides, scrub jungles of the hilly escarpments are useful in developing plantations (e.g. *Areca, Anacardium, Cacao, Casuarina, Cocos* and *Hevea*). Depending on the quality of laterite, some scrub jungles have been converted into quarries to extract stones. Due to relatively sparse vegetation in scrub jungles, exotic plant species, especially *Acacia* and *Lantana*, compete with native tree species such as *Careya arborea, Holigarna* sp., *Hopea ponga, Macaranga peltata, Sapium insigne, Syzygium cumini* and *Terminalia paniculata*. Dried grasses in scrub jungles during summer often succumb fire attack and such impacts on scrub jungles are low to medium and not as harsh as wild forest fire due to sparse vegetation.

Scrub jungles support numerous macrofungi owing to specific climatic conditions, phytogeographic set-up, accumulation of plant detritus and presence of termite mounds (Karun & Sridhar 2013; Greeshma et al. 2015). Although reports on the macrofungi in these scrub jungles are scanty, available reports from the west coast of India reveal occurrence of different groups of macrofungi (agarics, jelly fungi, polypores, puffballs, cup fungi, stinkhorns, xylarias and ectomycorrhizas) on different substrates (leaf/bark/woody litter, stubs, standing dead trees, soil and termite mounds) (Karun and Sridhar 2013, 2014a, 2014b; Sridhar & Karun 2013; Ghate et al. 2014; Ghate & Sridhar 2015a, 2015b; Pavithra et al. 2015). Some macrofungi occur in scrub jungles are edible (e.g. *Astraeus* sp., *Auricularia* sp., *Boletus* sp., *Lentinus* sp., *Lycoperdon* sp. and *Termitomyces* sp.), medicinal (e.g. *Daldinia* sp., *Ganoderma* sp., *Lentinus* sp., *Pycnoporus* sp. and *Xylaria* sp.) and ectomycorrhizal (e.g. *Amanita* sp., *Astraeus* sp., *Boletus* sp., *Geastrum* sp. and *Lycoperdon* sp.). Thus, further study of macrofungal assemblage, diversity and mutualistic association with plants/termites/insects in coastal region helps deriving future benefits through management strategies.

As an ecological factor, fire influences the atmosphere, soil, flora, fauna and microorganisms (McMullan-Fisher et al. 2011; Fischer et al. 2013; Kurth et al. 2013). Besides, fire is also responsible for complete or partial elimination of organic matter deposited on the soil depending on its intensity (low, medium and high) leading to indirect effect on fungal growth and perpetuation (Kennedy et al. 2015). Fire also influences soil physical (e.g. porosity, stability and water absorption), chemical (e.g. pH, nutrients status and C/N ratio) and biological (e.g. microbial composition, microbial biomass and mineral sequestration) properties (Doerr & Cerdà 2005; McMullan-Fisher et al. 2011). Impact of fire on fungi varies depending on several factors such as characteristics of soil, type of vegetation and intensity of fire (McMullan-Fisher et al. 2011; Kennedy et al. 2015). Ratkowsky and Gates (2009) have demonstrated succession of macrofungi related to time since fire in the lowland eucalypt forest of Southern Tasmania. Pyrophylic (fire-dependent) fungi are cosmopolitan, often fruit in large numbers and valuable in ecosystem recovery and restoration (Robinson et al. 2008; Bean et al. 2009; Claridge et al. 2009). According to Dahlberg (2002), in Swedish boreal forests, up to 40 species of pyrophylic fungi need postfire conditions for completion of their life cycles.

As the role of fungi in the recovery of forest ecosystems affected by fire is poorly understood, the major objective of the present study was to compare macrofungal assemblage and diversity in scrub jungles in control and fire-impacted regions. This study evaluates differences in macrofungal abundance based on species richness and sporocarp richness in relation to abiotic factors, similarity between sampling interval, substrate preference and economic importance.

## 2. Materials and methods

### 2.1. Scrub jungles

**Figure 1.**
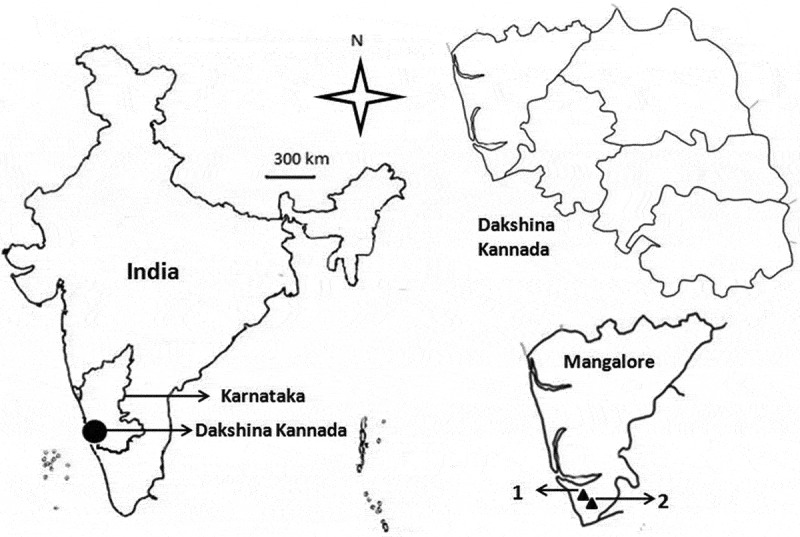
Map of the sampling site (1, control; 2, fire-impacted).

**Figure 2.**
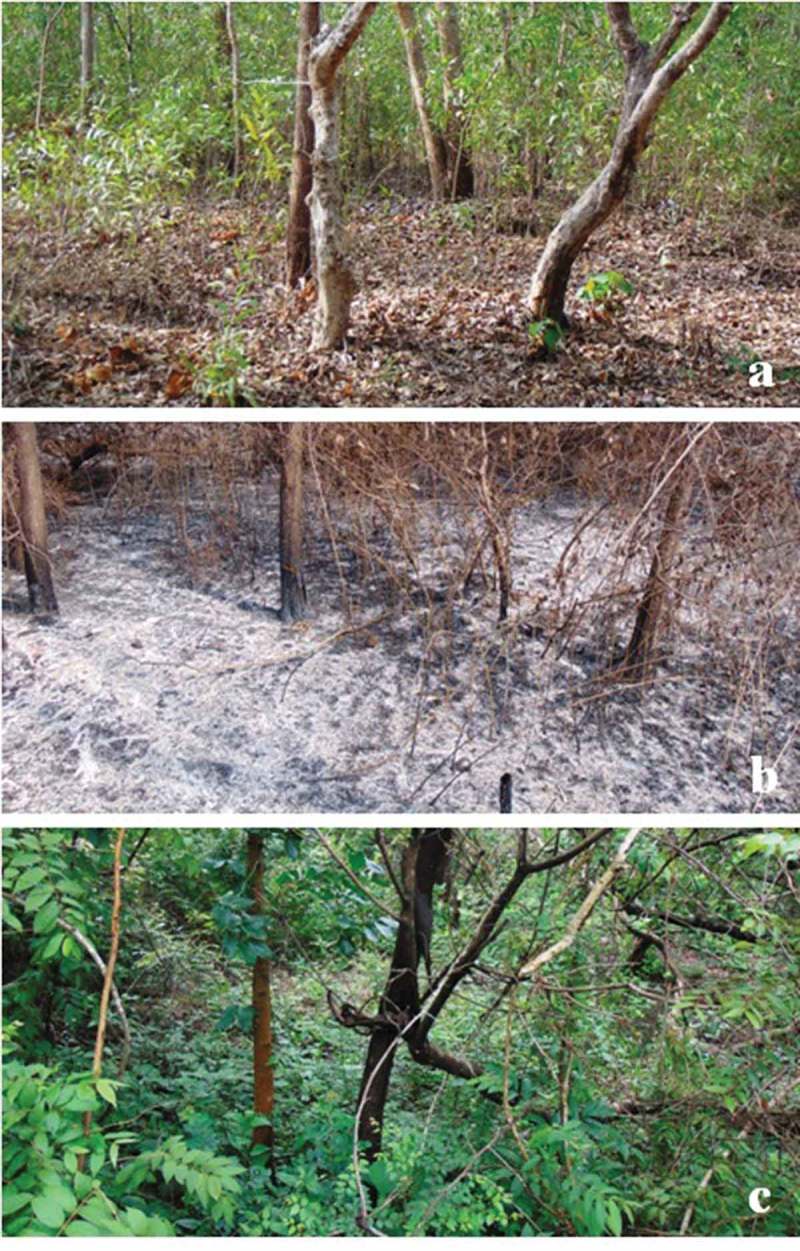
Representative locations of scrub jungle surveyed for macrofungi: Floor of a location during peak summer (a), floor of a location devastated by fire (b) and regenerated location of a floor during monsoon after fire impact (c).

Two scrub jungles on the lateritic hilly slopes (12°50ʹN, 74°60ʹE; 80–130 m asl) without fire impact (control) and with fire impact (impacted) during peak summer period (May 2014) were selected for survey (Figure 1). The control site consisting of a variety of herbs, shrubs and trees with grass bed, leaf, bark and woody litter (Figure 2a). The impacted site caught fire (accidental or deliberate), devastated, lost almost all understory biomass and resulted in thin spread of ash, charcoal, partially burnt medium and coarse woody litter without major impact on large tree trunks (Figure 2b). After fire attack during summer season, the scrub jungle recovered with good ground vegetation due to precipitation during monsoon (Figure 2c).

### 2.2. Macrofungi

Survey of macrofungi was carried out at fortnightly intervals on the onset of south-west monsoon (early June) up to 10 weeks (mid August 2014). On each sampling date, a quadrat (50 m^2^) was randomly chosen and surveyed for occurrence of sporocarps of macrofungi. Macromorphological characteristics of sporocarps were studied on the sampling site and representative samples were collected in sterile polythene bags to transfer to the laboratory. Micormorphological features were evaluated using high-power microscope (Nikon YS100, Japan) and identified using diagnostic keys (Pegler 1990; Jordan 2004; Phillips 2006; Cannon & Kirk 2007; Mohanan 2011; Buczacki 2012; Tibuhwa 2012; Karun and Sridhar 2013, 2014b). Selected macrofungi were blotted and preserved in a fixative (water–ethanol–formaldehyde: 14:5:1).

### 2.3. Abiotic factors

On each sampling date, abiotic features of air and soil were monitored from four corners of the quadrat chosen for survey. Air temperature (in shade) and soil temperature (~10 cm depth) were measured by a mercury thermometer (Model # 17876; ±0.28°C; N.S. Dimple Thermometers, New Delhi, India). Air humidity was measured using Digital Thermohygrometer (Model # TM-1; accuracy, ±1%; Mextech Technologies India Pvt, Ltd., Mumbai, India). The pH and electrical conductivity of soil were evaluated on dilution with distilled water (1:2.5 v/v) using water analysis kit (Model # 304; Systronics, Ahmedabad, India). To determine moisture (gravimetric method), organic carbon (Walkley and Black’s rapid titration method) and total nitrogen (macro-Kjeldahl method) of soil, protocols by Jackson (1973) were followed. Total phosphorus in soil was determined by vanadomolybdophosphoric acid method (AOAC 1990). The C/N ratio was calculated based on the quantities of organic carbon and total nitrogen.

### 2.4. Data analysis

The number of sporocarps of a species per quadrat (NSQ) during each fortnight (two weeks) was recorded for control as well as fire-impacted regions. The mean sporocarps per quadrat (MSQ) among five fortnights and per cent relative abundance (RA%) of each species were calculated. The Shannon’s diversity (Magurran 1988) and Pielou’s evenness (Pielou 1975) of macrofungi in each fortnight were determined. The Jaccard’s similarity (%) of macrofungi in each fortnight between control and fire-impacted regions was calculated according to Chao et al. (2005). The overall difference in abiotic factors of control and fire-impacted regions was evaluated by *t*-test (Statistica Version # 8 (StatSoft Inc. 2008). The Pearson correlation was employed to follow the relationship between species and sporocarp richness against 10 abiotic factors (*p*-values, two-tailed; confidence intervals, 95%) using SPSS 16.0 (www.spss.com).

## 3. Results

### 3.1. Spatial and temporal variation

**Figure 3.**
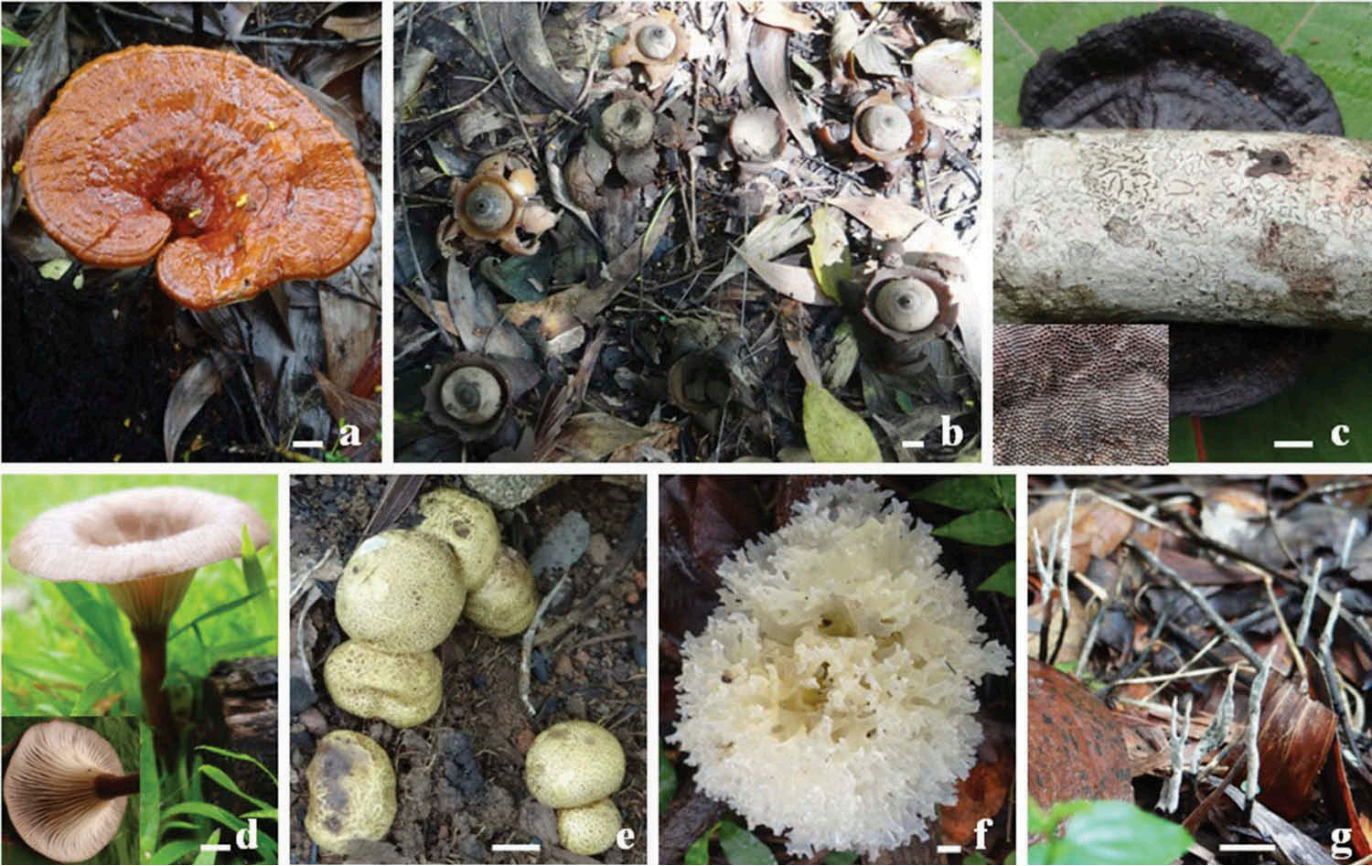
Representative macrofungi found in control region of scrub jungle: *Amauroderma conjunctum* on soil (a), *Geastrum triplex* on soil (b), *Hexagonia tenuis* on wood (inset, ventral side) (c), *Panus similis* on wood (inset, ventral side) (d), *Scleroderma citrinum* on soil (e), *Tremella reticulata* on wood (f) and *Xylaria hypoxylon* on wood (g) (scale bar, 1 cm).

**Figure 4.**
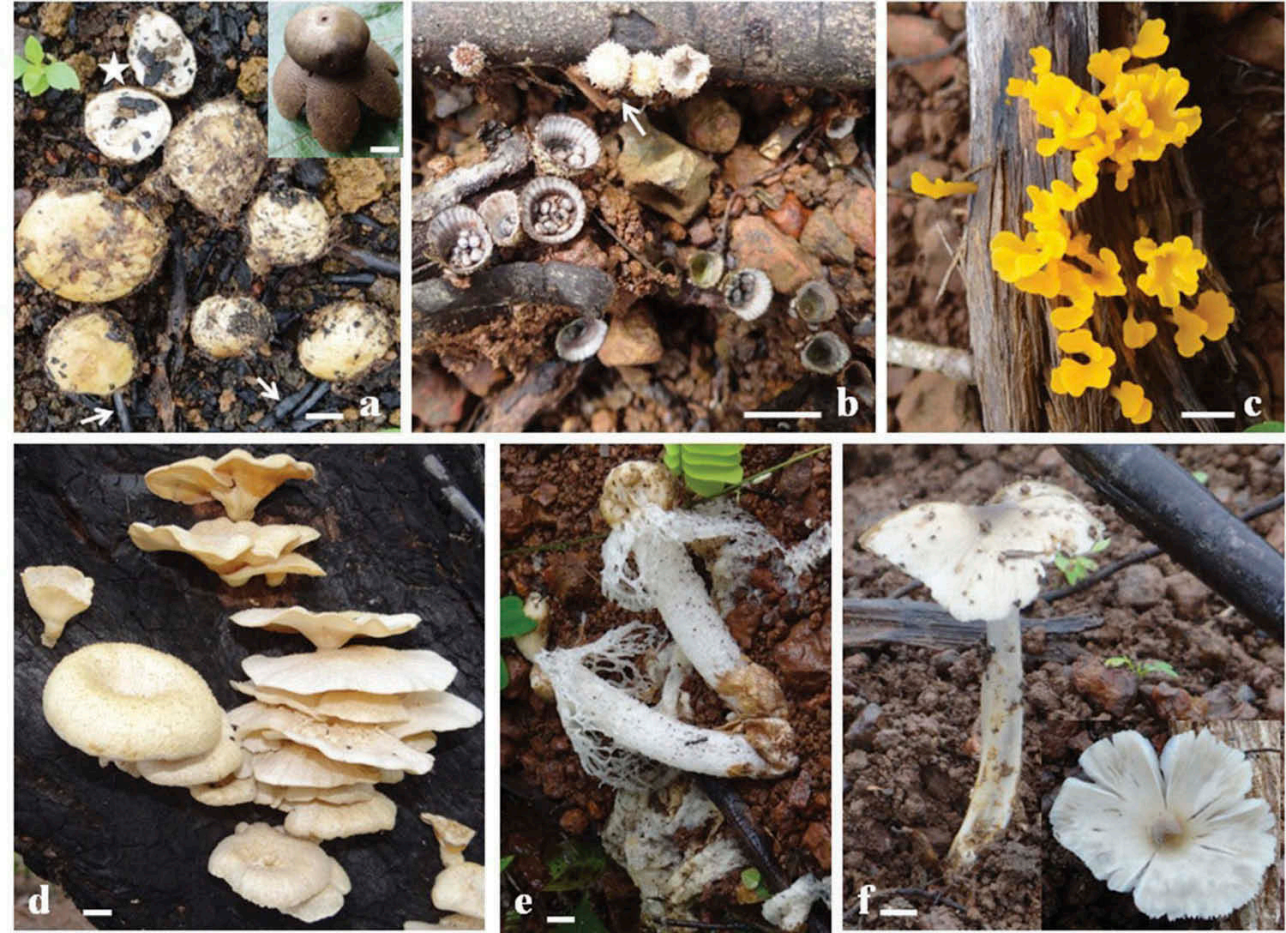
Representative macrofungi found in fire-impacted region of scrub jungle: Immature *Astraeus odoratus* on burnt soil (star, opened immature fruit body covered with charcoal pieces; arrows, charcoal pieces on burnt ground; inset, mature fruit body) (a), *Cyathus striatus* on burnt soil and wood (arrow, immature fruit bodies) (b), *Dacryopinax spathularia* on burnt wood (c), *Lentinus squarrosulus* on burnt wood (d), *Phallus indusiatus* on burnt soil (e) and *Termitomyces striatus* on burnt soil (inset, dorsal side showing distinct umbo) (f) (scale bar, 1 cm).

**Figure 5.**
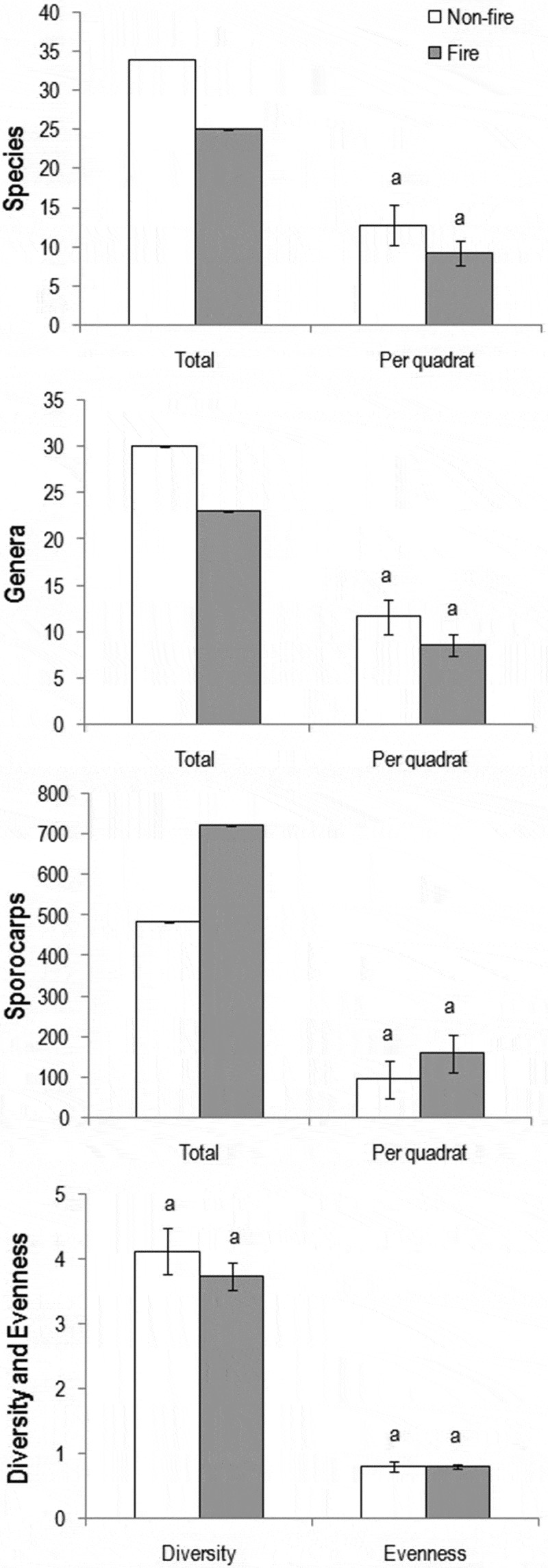
Total species, genera, sporocarps (with species, genera and sporocarps per quadrat), diversity and evenness of macrofungi found in control and fire-impacted regions of the scrub jungle (bars with same alphabet are not significantly differed: *p* > 0.05).

This inventory yielded 34 species (in 30 genera) and 25 species (in 23 genera) in the control and fire-impacted regions, respectively (Table 1; Figures 3 and 4). Although species as well as genera were higher in control than in fire-impacted region, they were not significantly different (Figure 5). Even though the sporocarp richness was higher in fire-impacted than in control region (723 vs. 484), it was not significantly different. Although the overall diversity and evenness were higher in control region, there was no significant difference.

**Table 1.** Occurrence of macrofungi in control and fire-impacted scrub jungles of the south-west coast of India.

Species	Number of sporocarps/ quadrat (50 × 50 m) in 2-week intervals (NSQ)					Mean sporocarps/quadrat (MSQ)	Relative abundance (RA%)	Substrate and importance
	2	4	6	8	10			
**Control scrub jungle**								
*Xylaria hypoxylon* (L.) Grev.	1	128	–	–	–	25.8	26.6	W**
*Thelephora palmata* (Scop.) Fr.	3	15	21	4	1	8.8	9.1	S***
*Marasmius* sp. 1	6	22	5	5	–	7.6	7.8	L
*Crepidotus* sp.	–	18	6	–	–	4.8	4.9	W
*Marasmius* sp. 2	8	16	–	–	–	4.8	4.9	L
*Geastrum triplex* Jungh.	–	–	–	–	23	4.6	4.7	S***
*Scleroderma citrinum* Pers.	6	9	–	4	–	3.8	3.9	S**, ***
***Marasmius* sp. 3**	4	9	5	–	–	3.6	3.7	L
*Clathrus delicatus* Berk. & Broome	–	15	–	–	–	3.0	3.1	W
***Lepiota s*p**.	–	3	5	6	1	3.0	3.1	S
*Marasmiellus* sp.	5	–	10	–	–	3.0	3.1	L
*Pluteus* sp.	12	3	–		–	3.0	3.1	S
*Lenzites* sp.	–	10	–	–	–	2.0	2.1	W
*Pisolithus albus* (Cooke & Massee) Priest	4	3	2	1	–	2.0	2.1	S***
*Microporus* sp.	1	4	3	–	–	1.6	1.7	W
***Schizophyllum commune* Fr**.	–	8	–	–	–	1.6	1.7	W
***Tetrapyrgos nigripes* (Fr.) E. Horak**	–	–	–	8	–	1.6	1.7	L
*Entoloma anamikum* Manim., A.V. Joseph & Leelav.	–	–	–	–	6	1.2	1.2	S
*Auricularia auricula-judae* (Bull.) Quél.	–	2	3	–	–	1.0	1.0	W*
*Clitocybe* sp.	–	–	5	–	–	1.0	1.0	S
*Hexagonia tenuis* Speg.	2	3	–	–	–	1.0	1.0	W
*Hygrocybe astatogala* (R. Heim) Heinem.	5	–	–	–	–	1.0	1.0	S***
*Phellinus* sp.	–	–	5		–	1.0	1.0	W
***Polyporus* sp**.	1	–	–	4	–	1.0	1.0	W
*Amauroderma conjunctum* (lloyd) Torrend	–	3	–	1	–	0.8	0.8	S**
*Hygrocybe aurantioalba* Leelav., Manim. & Arnolds	4	–	–	–	–	0.8	0.8	S***
*Panus similis* (Berk. & Broome) T.W. May & A.E. Wood	–	–	1	1	1	0.6	0.6	W
*Ramaria versatilis* Quél	3	–	–		–	0.6	0.6	S
*Tremella reticulata* (Berk.) Farl.	–	3	–	–	–	0.6	0.6	W*
*Amanita angustilamellata* (Höhn.) Boedijn	2	–	–	–	–	0.4	0.4	S***
*Clitocybe dealbata* (Sowerby) P. Kurmm.	2	–	–	–	–	0.4	0.4	S
*Ganoderma lucidum* (Curtis) P.Karst.	–	2	–	–	–	0.4	0.4	W**
***Chlorophyllum molybdites* (G. Mey.) Massee**	–	–	–	–	1	0.2	0.2	S
***Leucocoprinus birnbaumii* (Corda) Singer**	–	–	–	1	–	0.2	0.2	S
**Fire-impacted scrub jungle**								
*Cyathus striatus* (Huds.) Willd.	–	74	105	–	12	38.2	24.1	S, W
*Lentinus squarrosulus* Mont.	75	–	3	–	20	19.6	12.4	W*
*Mycena* sp. 1	–	–	21	53	–	14.8	9.3	S
*Astraeus odoratus* Phosri, Walting, M.P. Martin & Whalley	–	15	42	7	–	12.8	8.1	S*, ***
*Mycena* sp. 2	–	–	63	–	–	12.6	7.9	S
***Schizophyllum commune* Fr**.	16	34	–	–	2	10.4	6.6	W
*Dacryopinax spathularia* (Schwein.) G.W. Martin	–	13	12	21	–	9.2	5.8	W*
*Phallus indusiatus* Schltdl.	–	23	8	1	–	6.4	4.0	S*, **
***Chlorophyllum molybdites* (G. Mey.) Massee**	26	–	–	–	–	5.2	3.3	S
*Pycnoporus sanguineus* (L.) Murrill	–	–	23	–	–	4.6	2.9	W**
*Nectria cinnabarina* (Tode) Fr.	–	20	–	–	–	4.0	2.5	W
*Omphalotus olearius* (DC.) Singer	–	–	17	–	–	3.4	2.1	W
*Termitomyces striatus* (Beeli) R. Heim	–	5	4	1	6	3.2	2.0	S*
***Leucocoprinus birnbaumii* (Corda) Singer**	–	1	3	10	–	2.8	1.8	S
*Gymnopilus junonius* (Fr.) P.D. Orton	–	12	–	–	–	2.4	1.5	S
*Termitomyces clypeatus* R. Heim	–	–	5	6	–	2.2	1.4	S*
***Lepiota* sp**.	7	–	–	–	–	1.4	0.9	S
***Marasmius* sp. 3**	–	–	7	–	–	1.4	0.9	S
*Agaricus crocopeplus* Berk. & Broome	4	–	–	1	–	1.0	0.6	S
*Lycoperdon utriforme* Bull.	–	–	3	–	–	0.6	0.4	S*, ***
***Polyporus* sp**.	2	–	–	–	1	0.6	0.4	W
*Xylaria nigripes* (Klotzsch) Cooke	–	–	–	–	3	0.6	0.4	W**
*Daldinia concentrica* (Bolton) Ces. & De Not.	–	–	–	–	2	0.4	0.3	W**
***Tetrapyrgos nigripes* (Fr.) E. Horak**	–	–	–	2	–	0.4	0.3	W
*Trametes maxima* (Mont.) A. David & Rajchenb.	–	–	2	–	–	0.4	0.3	W

Notes: Common to control and fire-impacted regions are in bold. Substrate: L, leaf litter; S, soil; W, woody litter. Importance based on traditional knowledge: *, edible; **, medicinal; ***, ectomycorrhizal.

**Figure 6.**
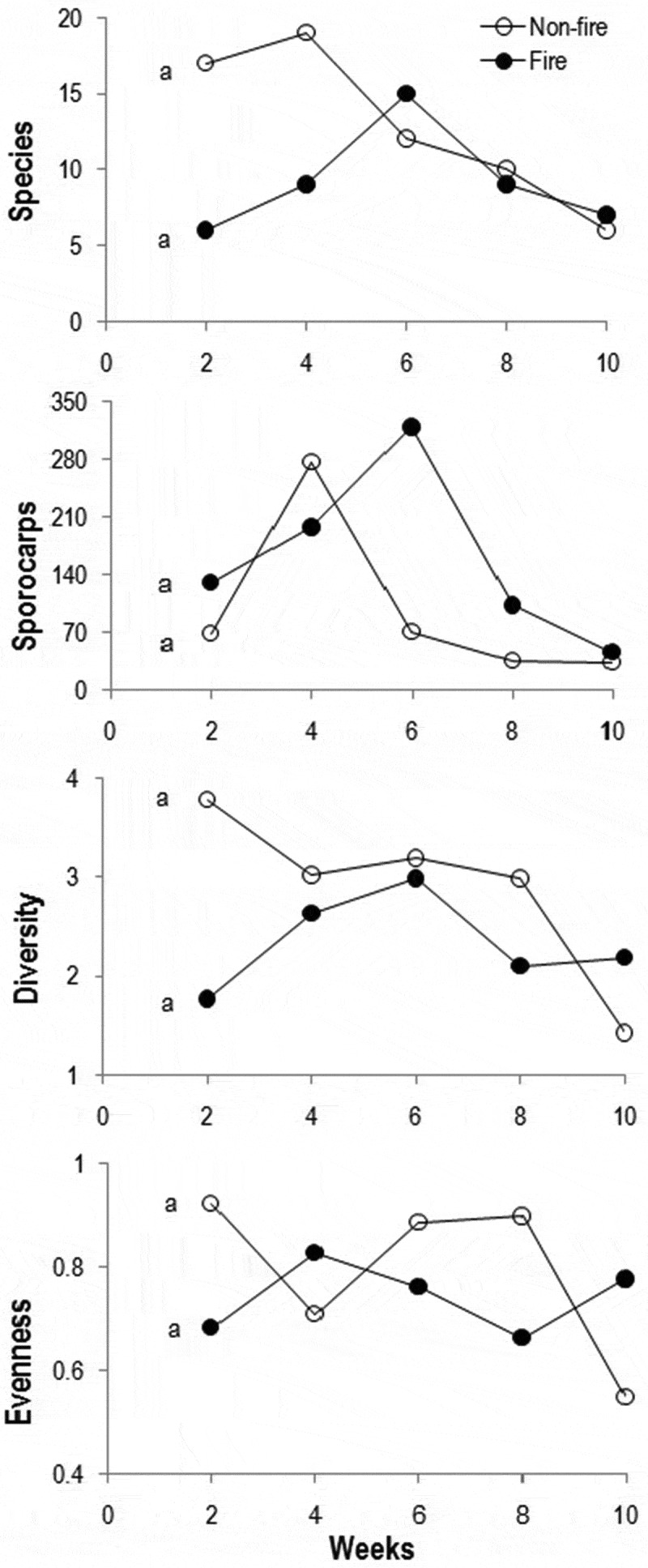
Fluctuation in number of species, sporocarps, diversity and evenness of macrofungi in control and fire-impacted regions of the scrub jungle (lines with same alphabet are not significantly differed: *p* > 0.05).

Among the five fortnights in control region, species (19 species) and sporocarp (276) richness attained the highest during the fourth week, while the Shannon diversity (3.779) on the second week (Figure 6). In fire-impacted region, the richness of species (15 species), sporocarp (318) and diversity (2.978) peaked during the sixth week. The overall Jaccard’s similarity between control and fire-impacted regions was higher (13.5%) than fortnights between regions (0–11.7%).

Among the 34 species recovered in control region, single-species dominance of *Xylaria hypoxylon* was seen with the highest relative abundance of 26.6% (Table 1). *X*. *hypoxylon* was highly dominant during the fourth week (128 sprorocarps/quadrat). *Thelephora palmata* was the second highest species (9.1%) occurred in all five samplings and 10 species were less abundant (<1%). In fire-impacted region, *Cyathus striatus* (24.1%) and *Lentinus squarrosulus* (32.4%) showed dominance (Table 1). Among them, *C. striatus* was most abundant during the sixth week (105 sporocarps/quadrat), while *L. squarrosulus* was abundant during the second week (75 sporocarps/quadrat). None of the species occurred in all fortnights and nine species were less abundant (<1%).

Seven species common to both regions include *Chlorophyllum molybdite, Lepiota* sp., *Leucocoprinus birnbaumii, Marasmius* sp. 3, *Polyporus* sp., *Schizophyllum commune* and *Tetrapyrgos nigripes*. The relative abundance of four species was higher in control region than in fire-impacted region (*Lepiota* sp., *Marasmius* sp. 3, *Polyporus* sp. and *T. nigripes*: 1–3.7 vs. 0.3–0.9%). In fire-impacted region, the relative abundance of *S. commune* was as high as 6.6% followed by *C. molybdite* (3.3%) and *L. birnbaumii* (1.8%), while the corresponding relative abundance in control region was 1.7%, 0.2% and 0.2%.

### 3.2. Substrate preference

**Figure 7.**
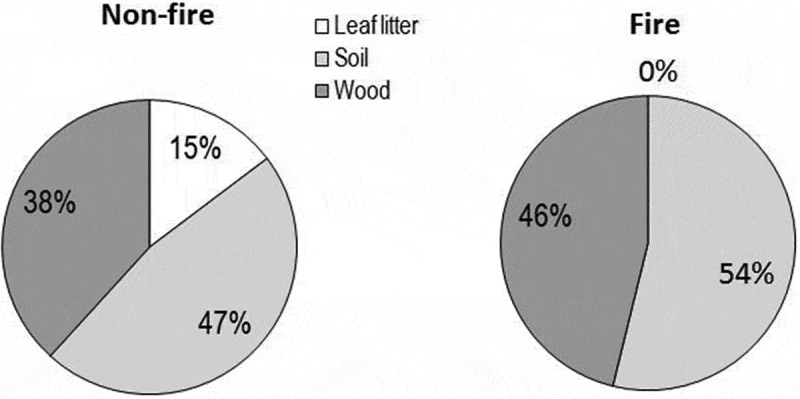
Per cent occurrence of macrofungi on leaf litter, soil and woody litter in control and fire-impacted regions of the scrub jungle.

A maximum of 16 species (47%) preferred soil, 13 species (38%) preferred woody litter and 5 species (15%) preferred leaf litter in control region (Table 1, Figure 7). In fire-impacted region, 13 species (54%) preferred burnt soil, 11 species (46%) preferred partially burnt woody litter and 1 species grew on burnt soil as well as partially burnt wood (*C*. *striatus*). Among the seven common species, the substrate preference of five species (*C. molybdite, Lepiota* sp., *L*. *birnbaumii, Polyporus* sp. and *S*. *commune*) was similar in both regions. The rest two species (*Marasmius* sp. 3 and *T*. *nigripes*) preferred leaf litter in control regions, while burnt soil in fire-impacted region.

### 3.3. Impact of abiotic factors

Except for air temperature, significant difference was seen in nine abiotic factors between control and fire-impacted region (Table 2). Air humidity was significantly lower in fire-impacted than in control region (*p* < 0.01). Soil temperature (*p *< 0.05), pH (*p* < 0.05), conductivity (*p *< 0.05), organic carbon content (*p* < 0.05), C/N ratio (*p* < 0.01) and total phosphorus content (*p* < 0.001) were significantly higher in fire-impacted than in control region, while it was opposite for soil moisture (*p *< 0.05) and total nitrogen content (*p* < 0.01). The Pearson correlation was negative between sporocarp richness and pH (*r* = −0.640) in control region, while the species richness was positively correlated with soil phosphorus content (*r* = 0.696) in fire-impacted region.

**Table 2.** Edaphic features of control and fire-impacted scrub jungles of south-west coast of India surveyed for macrofungi (mean, *n* = 20 ± SD).

	Air		Soil							
	Temperature (°C)	Humidity (%)	Temperature (°C)	pH	Conductivity (mS cm^−1^)	Moisture (%)	Organic carbon (%)	Total nitrogen (%)	C/N ratio	Total phosphorus (mg g^−1^)
Control	27.7 ± 1.3^a^	84.1 ± 8.2^a^	26.5 ± 0.9^a^	6.0 ± 0.6^a^	4.4 ± 1.0^a^	26.3 ± 3.0^a^	2.9 ± 0.5^a^	1.16 ± 0.2^a^	2.1 ± 0.7^a^	0.09 ± 0.01^a^
Fire-impacted	29.1 ± 1.6^a^	75.3 ± 6.0^b^**	28.4 ± 2.0^b^*	6.5 ± 0.7^b^*	12.8 ± 1.8^b^*	21.5 ± 4.4^b^*	6.6 ± 2.3^b^*	0.5 ± 0.1^b^**	14 ± 5.1^b^**	0.15 ± 0.01^b^***

Note: Values across the rows with different letters are significantly different, *t*-test: *p < 0.05; **p < 0.01; ***p < 0.001.

## 4. Discussion

The present study revealed occurrence of 52 species (in 45 genera) considering control and fire-impacted regions together with an overlap of seven species (13.5%). The macrofungal community in fire-impacted region differed drastically than control region as seen in burnt sites of eucalypt forests of the Western Australia (Robinson et al. 2008). Although the fire-impacted region in our study consists of less macrofungi (25 vs. 34 species), the total number of sporocarps was higher (723 vs. 484), possibly due to lack of competition by leaf litter fungi as well as fire-sensitive fungi. Overlap of 13.5% species between control and fire-impacted regions denotes facultative macrofungi. Interestingly, the relative abundance of three species was higher than control region (1.8–6.6% vs. 0.2–1.7%), while it was lower in the rest four species (0.3–0.9% vs. 1.3.7%) (see Table 1).

McMullan-Fisher et al. (2002) recognized three phases of fungal re-colonization during postfire conditions: (1) immediate phase (0 year), (2) intermediate phase (2–4 years) and (3) mature phase (7 years). Such clear-cut phases seem to be dependent on the type of forest and intensity of fire. Macrofungi found in fire-impacted region of the scrub jungle have the capacity to overcome the effect of fire in different ways (mycelia residing deep in soil, deep in exposed woody litter and endophytic in below ground roots). For instance, endophytic *Daldinia* spp. and *Hypoxylon* spp. thrive within the wood of healthy trees and shrubs and produce visible fruit bodies on host senescence or host wood affected by fire (Robinson et al. 2008). Partially burnt wood inhabiting *Daldinia loculata* in our study was confined to dead birch trees in postfire due to presence of latent mycelia (Johannesson et al. 2001). Increase in fruiting of *Daldinia* spp. was seen in fire-affected forests in Tasmania and Western Australia (Gates et al. 2005; Robinson et al. 2008). Besides, some fungi showed increased heat resistance of mycelia, spores and sclerotia as adaptation to forest fire (Barr et al. 1999; Suryanarayanan et al. 2011). Egger and Paden (1986) designated some postfire fungi as carbonicolous as they fruit on heated soil, partially burnt organic debris, charcoal and ash.

Impact of fire on ectomycorrhizal communities showed neutral, positive and negative effects (e.g. Barr et al. 1999; Mah et al. 2001; Chai et al. 2013; Kennedy et al. 2015). No significant effect of fire was seen on ectomycorrhizal fungi in broadcast-burned clearcuts in British Columbia (Mah et al. 2001). Kennedy et al. (2015) demonstrated that forest soil type in British Columbia have greater influence than severity of fire on ectomycorrhizal communities. Although heating the soil samples collected from three depths from Scots pine forest stand in Leuk, Valais (45°C, 60°C and 70°C) reduced ectomycorrhizal species at 60°C and 70°C, some species survived heating (Kipfer et al. 2010). Interestingly, low-intensity fire in site management facilitated seed germination and seedling establishment although limits ectomycorrhizal diversity up to some extent in forests of British Columbia (Wiensczyk et al. 2002). The role of ectomycorrhizae and fungal mycelial mats in soil are highly valuable in nutrient acquisition (as nutrients leach out by rains) and stabilization (by soil aggregation) in sloppy scrub jungles. However, low-intense pre-monsoon showers likely help selected macrofungal growth, sequester nutrients and restore soil qualities. As porous charcoal adsorb water similar to soil strata (Pietikainen et al. 2000), those fungi hidden within partially charred woody litter will be benefitted. During postfire conditions, significant decrease in mycorrhizal species was seen in *Cistus* and *Pinus* plots of Spain (Martín-Pinto et al. 2006), corroborating with the present study. The control region of scrub jungle consists of seven (*Amanita angustilamellata, Geastrum triplex, Hygrocybe astatogala, H*. *aurantioalba, Pisolithus albus, Scleroderma citrinum* and *T*. *palmata*), while the fire-impacted region consists of two (*Astraeus odoratus* and *Lycoperdon utriforme*) ectomycorrhizal fungi and it is likely that the latter two species are important in soil rejuvenation. Similarly, other dominant macrofungi in fire-impacted region (e.g. *A*. *odoratus*, *C*. *striatus*, *L*. *squarrosulus, Mycena* spp. and *S*. *commune*) are also valuable in soil rejuvenation. The fire-damaged cashew plantation adjacent to the scrub jungle showed preponderance of *Gymnopilus* sp. on partially burnt woody litter (Karun & Sridhar 2014a). In bamboo thickets, *Gymnopilus junonius* was predominant on the burnt soil as well as on partially burnt wood (Karun et al. 2014). Although *G. junonius* was not dominant in our study, it was restricted to burnt soil in fire-impacted region. The dominant *S. commune* on burnt wood in our study is also common on burnt wood on the coastal sand dunes of south-west India (K.R. Sridhar, unpub. obs.). Mycorrhizal *L. utriforme* was abundant in coastal sand dunes during postfire conditions. Edible and ectomycorrhizal *A*. *odoratus* dominated in fire-impacted scrub jungle (Pavithra et al. 2015). In the Northern Thailand, burnt floors of dipterocarp-oak forests showed significant increase in the yield of *A. odoratus* (Kennedy et al. 2012). Sysouphanthong et al. (2010) opined that *Astraeus hygrometricus* was stimulated by fire in tea plantations of Thailand. Another ectomycorrhizal fungus, *G. triplex*, was also often associated with native tree species of scrub jungles in south-west coast of India (Karun & Sridhar 2014a). However, according to Sysouphanthong et al. (2010), the yield of edible macrofungi will be lowered in those forests due to fire damage.

The period and interval of survey of an ecosystem are important aspects in documentation of macrofungi. Karun (2014) followed monthly intervals in the Western Ghats as south-west monsoon persists longer than south-west coastal region. Due to less vegetation, dry conditions and porous lateritic bed in the south-west India, there are chances to miss many macrofungi on monthly survey and thus weekly or fortnightly surveys would be preferable. Unlike control region (peak in species and sporocarps, fourth week; peak in diversity, second week), fire-impacted region required 2–4 weeks to recover and attain the highest species, sporocarps and diversity (sixth week). Subsequent decrease in species was almost similar in both regions, but the recession of sporocarps was slower in fire-impacted than in control region.

There seems to be cumulative effect of abiotic factors on the richness, diversity and distribution of macrofungi in scrub jungles. Except for air temperature, rest of the nine abiotic factors between control and fire-impacted regions differed significantly. In fire-impacted region, the species richness was positively correlated with soil phosphorus content, which was considerably higher than control region (0.15 vs. 0.09 mg g^−1^). There was a negative correlation of sporocarp richness against pH in control region, which was acidic than fire-impacted region (pH, 6 vs. 6.5). The total nitrogen content in soils of fire-impacted region was significantly lower compared to control region (0.5% vs. 1.2%). However, Claridge et al. (2009) opined that some fungi have the capacity to capture newly released and highly leachable nitrogen ions by converting into organic compounds necessary for their growth and sporulation.

**Figure 8.**
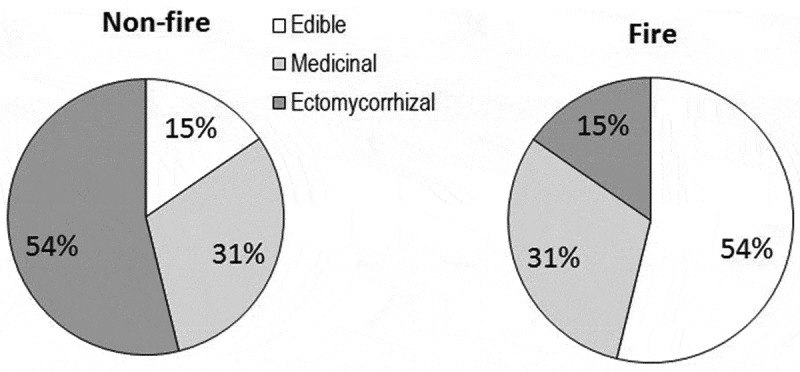
Per cent occurrence of edible, medicinal and ectomycorrhizal fungi in control and fire-impacted regions of the scrub jungle.

Compared to control region, many fragile fungi were not found in the fire-impacted region (e.g. *Marasmius, Marasmiellus* and *Entoloma*). However, *Marasmius* sp. 3 was common to both regions and grew on the soil in fire-impacted region possibly due to lack of leaf litter. Our study revealed occurrence of 12 (34%) and 10 (42%) species of macrofungi as edible (15% and 54%), medicinal (31% each) and ectomycorrhizal (54% and 15%) in control and fire-impacted regions, respectively (Figure 8). Some studies revealed that fruit body production in selected macrofungi will be stimulated by fire (Carpenter et al. 1987; Duchesne & Weber 1993). According to Dahlberg (2002), some ectomycorrhizal fungi have adapted to low and high intensity of postfire conditions. Based on the traditional knowledge, the fire-impacted region yielded seven edible fungi against two in control region. *L*. *squarrosulus* is a second dominant fungus in fire-impacted region and fruiting of two termitomycetes (*Termitomyces clypeatus* and *T. striatus*) in fire-impacted region seems to be stimulated by fire. Besides these, other termitomycetes (*Termitomyces fulginosus, T. microcarpus, T. schimperi* and *T. umkowaan*) were also recently recorded from the scrub jungles (Karun & Sridhar 2013, 2014a; Ghate et al. 2014; K.R. Sridhar, unpub. obs.).

The present study demonstrated a drastic difference in macrofungal assemblage and diversity based on the impact of fire in scrub jungles of the south-west India. Due to human interference, the scrub jungles became fragile ecosystem and depletion of economically valuable native tree species such as *C. arborea, H. ponga* and *T. paniculata* may adversely affect macrofungal communities. Many questions need to be addressed on the macrofungi in scrub jungles in future: (1) Are macrofungi in fire-impacted regions recover during subsequent years similar to control regions? (2) Is there any succession of macrofungi after fire impact? (3) What happens to macrofungi if scrub jungles are repeatedly attacked by fire? Further studies on the impact of fire on community composition, species interaction and life history of macrofungi are rewarding to impart management strategies.

## References

[CIT0001] AOAC 1990 Official methods of analysis. 15th ed. Washington (DC): Association of Official Analytical Chemists.

[CIT0002] Baar J, Horton TR, Kretzer AM, Bruns TD. 1999 Mycorrhizal colonization of *Pinus muricata* from resistant propagules after a stand-replacing wildfire. New Phytol. 143:409–418.

[CIT0003] Bean L, Kostow N, Tempest A 2009 The effects of prescribed burning on macrofungal species richness in upland, white oak forest. Tillers. 6:27–32.

[CIT0004] Buczacki S 2012 Collins fungi guide. London: Harper-Collins Publishers.

[CIT0005] Cannon PF, Kirk PM 2007 Fungal families of the world. Wallingford: CAB International.

[CIT0006] Carpenter SE, Trappe JM, Ammirati J 1987 Observations of fungal succession in the Mount St. Helens devastation zone, 1980–1983. Can J Bot. 65:716–728.

[CIT0007] Chai D–D, Guo S–J, Sun X–B, Qin T–T 2013 The major factors affecting ectomycorrhizal fungi diversity in the forest ecosystem. Adv J Food Sci Technol. 5:879–890.

[CIT0008] Chao A, Chazdon RL, Colwell RK, Shen T-J 2005 A new statistical approach for assessing similarity of species composition with incidence and abundance data. Ecol Lett. 8:148–159.

[CIT0009] Claridge AW, Trappe JM, Hansen K 2009 Do fungi have a role as soil stabilizers and remediators after forest fire? For Ecol Manage. 257:1063–1069.

[CIT0010] Dahlberg A 2002 Effects of fire on ectomycorrhizal fungi in Fennoscandian boreal forests. Silva Fennica. 36:69–80.

[CIT0011] Doerr SH, Cerdà A 2005 Fire effects on soil system functioning: new insights and future challenges. Int J Wildland Fire. 14:339–342.

[CIT0012] Duchesne LC, Weber MG 1993 High incidence of the edible morel *Morchella conica* in a jack pine, *Pinus banksiana*, forest following prescribed burning. Can Field Natur. 107:114–116.

[CIT0013] Egger KN, Paden JW 1986 Biotrophic associations between lodgepole pine seedlings and postfire ascomycetes (Pezizales) in monoxenic culture. Can J Bot. 64:2719–2725.

[CIT0014] Fischer A, Marshall P, Camp A 2013 Disturbances in deciduous temperate forest ecosystems of the northern hemisphere: their effects on both recent and future forest development. Biodivrs Conserv. 22:1863–1893.

[CIT0015] Gates GM, Ratkowsky DA, Grove SJ 2005 A comparison of macrofungi in young silvicultural regeneration and mature forest at the Warra LTER site in the southern forests of Tasmania. Tasforests. 16:127–152.

[CIT0016] Ghate SD, Sridhar KR 2015a Contribution to the knowledge on macrofungi in mangroves of the southwest India. Plant Biosys. doi:10.1080/11263504.2014.994578

[CIT0017] Ghate SD, Sridhar KR 2015b Macrofungi in coastal sand dunes and mangroves of southwest India In: Pullaiah T, Rani S, editors. Biodiversity in India. Vol. 8 New Delhi: Regency Publications; p. 229–246.

[CIT0018] Ghate SD, Sridhar KR, Karun NC 2014 Macrofungi on the coastal sand dunes of south-western India. Mycosphere. 5:144–151.

[CIT0019] Greeshma AA, Sridhar KR, Pavithra M 2015 Macrofungi in the lateritic scrub jungles of southwestern India. J Threatened Taxa. 7:7812–7820.

[CIT0020] Jackson ML 1973 Soil chemical analysis. New Delhi: Prentice Hall of India.

[CIT0021] Johannesson H, Vasiliauska R, Dahlberg A, Penttilä R, Stenlid J 2001 Genetic differentiation in Eurasian populations of the post-fire ascomycete Daldinia loculata. Mol Ecol. 10:1665–1677. 11472535 10.1046/j.1365-294x.2001.01317.x

[CIT0022] Jordan M 2004 The encyclopedia of fungi of Britain and Europe. London: Francis Lincoln.

[CIT0023] Karun NC 2014 Studies on macrofungi and aquatic hyphomycees of the Western Ghats and west coast of India [PhD thesis in Biosciences]. India: Mangalore University.

[CIT0024] Karun NC, Sridhar KR 2013 Occurrence and distribution of *Termitomyces* (Basidiomycota, Agaricales) in the Western Ghats and on the west coast of India. Czech Mycol. 65:233–254.

[CIT0025] Karun NC, Sridhar KR 2014a A preliminary study on macrofungal diversity in an arboretum and three plantations of the southwest coast of India. Curr Res Environ Appl Mycol. 4:173–187.

[CIT0026] Karun NC, Sridhar KR 2014b Geasters in the Western Ghats and west coast of India. Acta Mycol. 49:207–219.

[CIT0027] Karun NC, Sridhar KR, Appaiah KAA 2014 Diversity and distribution of macrofungi in Kodagu region (Western Ghats) – a preliminary account In: Pullaiah T, Karuppusamy S, Rani S, editors. Biodiversity in India. Vol. 7 New Delhi: Regency Publications; p. 73–96.

[CIT0028] Kennedy KH, Maxwell JF, Lumyong S 2012 Fire and the production of *Astraeus odoratus* (Basidiomycetes) sporocarps in deciduous dipterocarp-oak forests of northern Thailand. Maejo Int J Sci Technol. 6:483–504.

[CIT0029] Kennedy NM, Robertson SJ, Green DS, Scholefield SR, Arocena JM, Tackaberry LE, Massicotte HB, Egger KN 2015 Site properties have a stronger influence than fire severity on ectomycorrhizal fungi and associated N-cycling bacteria in regenerating post-beetle-killed lodgepole pine forests. Folia Microbiol. 60:399–410. 25540132 10.1007/s12223-014-0374-7

[CIT0030] Kipfer T, Egli S, Ghazoul J, Moser B, Wohlgemuth T 2010 Susceptibility of ectomycorrhizal fungi to soil heating. Fungal Biol. 114:467–472. 20943157 10.1016/j.funbio.2010.03.008

[CIT0031] Kurth VJ, Fransioli N, Fule PZ, Hart SC, Gehring CA 2013 Stand-replacing wildfires alter the community structure of wood-inhabiting fungi in southwestern ponderosa pine forests of the USA. Fungal Ecol. 6:192–204.

[CIT0032] Magurran AE 1988 Ecological diversity and its measurement. Princeton (NJ): Princeton University Press.

[CIT0033] Mah K, Tackaberry LE, Egger KN, Massicotte HB 2001 The impacts of broadcast burning after clear-cutting on the diversity of ectomycorrhizal fungi associated with hybrid spruce seedlings in central British Columbia. Can J Forest Res. 31:224–235.

[CIT0034] Martín-Pinto P, Vaquerizo H, Peñalver F, Olaizola J, Andrés J, Oria-de-Rueda JA 2006 Early effects of a wildfire on the diversity and production of fungal communities in Mediterranean vegetation types dominated by *Cistus ladanifer* and *Pinus pinaster* in Spain. For Ecol Manage. 225:296–305.

[CIT0035] McMullan-Fisher SJM, May TW, Keane PJ 2002 The macrofungal community and fire in a Mountain Ash forest in southern Australia In: Hyde KD, Jones EBG, editors. Fungal succession. Fungal diversity research series # 10. Hong Kong: Hong Kong University Press; p. 57–76.

[CIT0036] McMullan-Fisher SJM, May TW, Robinson RM, Bell TL, Lebel T, Catcheside P, York A 2011 Fungi and fire in Australian ecosystems: a review of current knowledge, management implications and future directions. Aust J Bot. 59:70–90.

[CIT0037] Mohanan C 2011 Macrofungi of Kerala. Handbook # 27. Peechi: Kerala Forest Research Institute.

[CIT0038] Pavithra M, Greeshma AA, Karun NC, Sridhar KR 2015 Observations on the *Astraeus* spp. of southwestern India. Mycosphere. 6:421–432.

[CIT0039] Pegler D 1990 Kingfisher field guide to the mushrooms and toadstools of Britain and Europe. London: Kingfisher Publications.

[CIT0040] Phillips R 2006 Mushrooms. London: Pan Macmillan.

[CIT0041] Pielou FD 1975 Ecological diversity. New York (NY): Wiley InterScience.

[CIT0042] Pietikainen J, Kiikkila O, Fritze H 2000 Charcoal as a habitat for microbes and its effect on the microbial community of the underlying humus. Oikos. 89:231–242.

[CIT0043] Ratkowsky DA, Gates GM 2009 Macrofungi in early stages of forest regeneration in Tasmania’s southern forests. Tasforests. 18:55–66.

[CIT0044] Robinson RM, Mellican AE, Smith RH 2008 Epigeous macrofungal succession in the first five years following a wildfire in karri (*Eucalyptus diversicolor*) regrowth forest in Western Australia. Aust Ecol. 33:807–820.

[CIT0045] Sridhar KR, Karun NC 2013 On the basket stinkhorn mushroom *Phallus merulinus* (Phallaceae) in Mangalore, Karnataka, India. J Threatened Taxa. 5:3985–3988.

[CIT0046] StatSoft 2008 Statistica, version # 8. Oklahoma: StatSoft Inc.

[CIT0047] Suryanarayanan TS, Govindarajulu MB, Thirumalai E, Reddy MS, Money NP 2011 Agni’s fungi: heat-resistant spores from the Western Ghats, southern India. Fungal Ecol. 115:833–838. 10.1016/j.funbio.2011.06.011 21872180

[CIT0048] Sysouphanthong P, Thongkantha S, Zhao R, Soytong K, Hyde KD 2010 Mushroom diversity in sustainable shade tea forest and the effect of fire damage. Biodivers Conser. 19:1401–1415.

[CIT0049] Tibuhwa DD 2012 *Termitomyces* species from Tanzania, their cultural properties and unequalled basidiospores. J Biol Life Sci. 3:140–159.

[CIT0050] Wiensczyk AM, Gamiet S, Durall DM, Jones MD, Simard SW 2002 Ectomycorrhizae and forestry in British Columbia: a summary of current research and conservation strategies. B C J Ecosys Manage. 2:1–20.

